# Determination of copy number and circularization ratio of Tn*916*-Tn*1545* family of conjugative transposons in oral streptococci by droplet digital PCR

**DOI:** 10.1080/20002297.2018.1552060

**Published:** 2018-12-06

**Authors:** Tracy Munthali Lunde, Adam P. Roberts, Mohammed Al-Haroni

**Affiliations:** a Department of Clinical Dentistry, Faculty of Health Sciences, UiT the Arctic University of Norway, Tromsø, Norway; b Department of Parasitology, Liverpool School of Tropical Medicine, Liverpool, UK; c Centre for Drugs and Diagnostics, Liverpool School of Tropical Medicine, Liverpool, UK

**Keywords:** Oral streptococci, Tn*916*-Tn*1545* family, antibiotic resistance, mobile genetic elements (MGEs), droplet digital PCR

## Abstract

**Background:** Tn916 and Tn1545 are paradigms of a large family of related, broad host range, conjugative transposons that are widely distributed in bacteria and contribute to the spread of antibiotic resistance genes (ARGs). Variation in the copy number (CN) of Tn916-Tn1545 elements and the circularization ratio (CR) may play an important role in propagation of ARGs carried by these elements.

**Objectives and Design:** In this study, the CN and CR of Tn916-Tn1545 elements in oral streptococci were determined using droplet digital PCR (ddPCR). In addition, we investigated the influence of tetracycline on the CR of Tn916-Tn1545 elements.

**Results:** The ddPCR assay designed in this study is a reliable way to rapidly determine CN and CR of Tn916-Tn1545 elements.

**Conclusions:** Our data also suggest that Tn916-Tn1545 elements are generally stable without selective pressure in the clinical oral Streptococcus strains investigated in this study.

## Introduction

The oral cavity is among the most microbiologically diverse environments in the human body and has been shown to contain over 1100 different bacterial species [1] of which *Streptococcus* species are the most abundant [2]. Although the majority of the *Streptococcus* species are not considered pathogenic, some species such as *Streptococcus mutans* are responsible for oral diseases and others, such as viridans group streptococci, can cause infections (such as pneumonia, endocarditis, and intra-abdominal infection) at other body sites [3].

There has been an increase in the number of antibiotic-resistant streptococcal strains over the last few decades [4], and recent studies suggest that the oral cavity functions as a reservoir for transferable antibiotic resistance genes [5–8] including genes encoding resistance to macrolides [9], beta-lactams, and tetracyclines [10]. One of the most common tetracycline resistance genes within oral isolates and metagenomes is *tet*(M) [8,11]. The broad distribution of *tet*(M) has frequently been linked to its association with mobile genetic elements (MGE) from the Tn*916-*Tn*1545* family of conjugative transposons/Integrative Conjugative Elements [ICEs] [12–16].

### The *Tn*916 conjugative transposons/ICE

Tn*916* (accession number; U09422.1) is an 18-kb broad host range ICE [17] first isolated from *Enterococcus faecalis* DS16 [18]. Tn*916* contains 24 ORFs (open reading frames) which are arranged in functional modules. These modules are responsible for conjugal transfer, transcriptional regulation, excision and insertion reactions (transposition), and accessory functions such as antibiotic and antiseptic resistance [17]. The transfer of Tn*916* from a donor cell to a recipient cell involves the excision of the element from its original replicon to form a circular intermediate (CI) molecule [19], which has also recently been shown to autonomously replicate [20]. Tn*916* and many related elements of the Tn*916*-Tn*1545* family are frequently able to insert into multiple sites within a host genome [21].

Variations in ICE CN (copy number) may have an impact on their stability (of the ICE and the host genome) and conjugation potential thereby influencing the level of antibiotic resistance within bacterial populations [22–24].

Evaluation of the CN of the Tn*916*-Tn*1545*-like elements in oral streptococci can be achieved by Restriction Fragment Length Polymorphism (RFLP) followed by southern blot hybridization [25], full genome sequencing and assembly [26], and by real-time quantitative PCR (qPCR) [20]. The first two methods are not only time consuming and labor intensive but they also require high quantities of pure, high molecular weight DNA. The qPCR has become a common method in determining CN of target genes [27], however it does have some limitations such as performance variation in and between assays [28] and artificial qPCR data resulting from samples with low target concentration but high levels of impurity [29]. These limitations can be overcome by droplet digital PCR (ddPCR) as it has been shown to produce more precise and reproducible results when compared to qPCR [30]. In the QX200 ddPCR (Bio-Rad, USA), a single PCR reaction is divided into approximately 20,000 droplets which are treated as individual reactions. Each reaction contains the relevant forward and reverse primer, the probes to detect the target gene and template DNA molecule.

In this study, we present an assay that rapidly reports CN of integrated and CI of Tn*916*-Tn*1545*-like elements in various clinical oral *Streptococcus* species. In addition, the CR of CI in the study strains is reported (percentage of CI molecules detected within the bacterial population as a function of the total number of host genomes).

## Materials and method

### Control strains

The fully sequenced *Bacillus subtilis* BS34A (NZ_LN680001.1), *B. subtilis* BS49 (NZ_LN649259.1), *Enterococcus faecium* OrEc1, and *E. faecium* OrEc2 derivatives containing different CN of Tn*916* were used as control strains in ddPCR (Table 1).

**Table 1.** Bacterial strains used in this study.

Bacteria	Relevant properties (MIC Tet)	Reference or Source
***B. subtilis* BS34** (Control strain containing one copy of Tn*916*)	*Tetracycline resistant bacterium (32*μg/ml)	[26]
***B. subtilis BS49*** (Control strain containing two copies of Tn*916*)	*Tetracycline resistant bacterium(48*μg/ml)	[26]
***E. faecium* OrEc1** (Control strain containing five copies of Tn*916*)	*Tetracycline resistant (96*μg/ml) (Transconjugant)	This study
***E. faecium* OrEc2** (Control strain containing one copy of Tn*916*)	*Tetracycline resistant (48*μg/ml) (Transconjugant)	This study
***S. pneumonia*** (control strain for Tetracycline MIC)	*Tetracycline susceptible* (≤1μg/ml)	ATCC 49619
***S. mitis SM2*8**	*Tetracycline resistant* clinical isolate *(64*μg/ml)	This study
***S. mitis SM2*9**	*Tetracycline resistant* clinical isolate *(32*μg/ml)	This study
***S. sanguinus* SS33**	*Tetracycline resistant* clinical isolate *(24*μg/ml)	This study
***S. sanguinus* SS41**	*Tetracycline resistant* clinical isolate *(32*μg/ml)	This study
***S. oralis* SO44**	*Tetracycline resistant* clinical isolate *(24*μg/ml)	This study
***S. oralis* SO47**	*Tetracycline resistant* clinical isolate (32μg/ml)	This study
***S. oralis* SO62**	*Tetracycline resistant* clinical isolate (4μg/ml)	This study
***S. oralis* SO67**	*Tetracycline resistant* clinical isolate (48μg/ml)	This study
***S. gordonii* SG71**	*Tetracycline resistant* clinical isolate (32μg/ml)	This study
***S. oralis* SO74**	*Tetracycline susceptible* clinical isolate (2μg/ml)	This study

### Clinical oral streptococcus strains

A selection of 10 antibiotic resistant oral *Streptococcus* strains collected by the National Advisory Unit for Detection of Antimicrobial Resistance (K-RES), University Hospital of North Norway were used in this study. These 10 strains tested PCR positive for *tet*(M) and the Tn*916* integrase (i*ntTn*) and excisionase (*xisTn*) genes. The 10 strains were further identified at the species level using MALDI-TOF. MALDI-TOF identification was carried out at the National Advisory Unit for Detection of Antimicrobial Resistance (K-RES) laboratories, University Hospital of North Norway. These strains were used in the determination of CN and CR of Tn*916*-Tn*1545* like elements in oral streptococci.

### Bacterial cultivation

*B. subtilis* and *E. faecium* strains were cultivated on Luria-Bertani (LB) agar at 37°C under aerobic conditions whereas the oral *Streptococcus* strains (Table 1) were cultivated in anaerobic conditions on Todd Hewitt (TH) agar at 37°C overnight using the Anaerocult® System (Merck, Germany).

### Determination of tetracycline MIC

The MIC of tetracycline for the oral streptococci was determined by E-test (BioMerieux, France) on Mueller-Hinton agar supplemented with 5% sheep blood and interpreted according to the European Committee on Antimicrobial Susceptibility Testing (EUCAST) (www.eucast.org). The *S. pneumoniae* ATCC 49619 was included in all the runs as a positive control.

### DNA extraction and DNA concentration measurement

The QIAcube (Qiagen, Hilden, Germany) automated system was used to extract DNA with a preprogramed protocol using the QIAamp DNA Mini Kit (Qiagen, Germany) to obtain DNA from all bacterial strains in this study according to the manufacturer’s instructions. The quality and yield of extracted genomic DNA were analyzed by agarose gel electrophoresis before determining the DNA concentration with the Qubit 3.0 fluorometer (Life Technologies, USA) according to the manufacturer’s instructions.

### Conventional PCR

**Figure 1.**
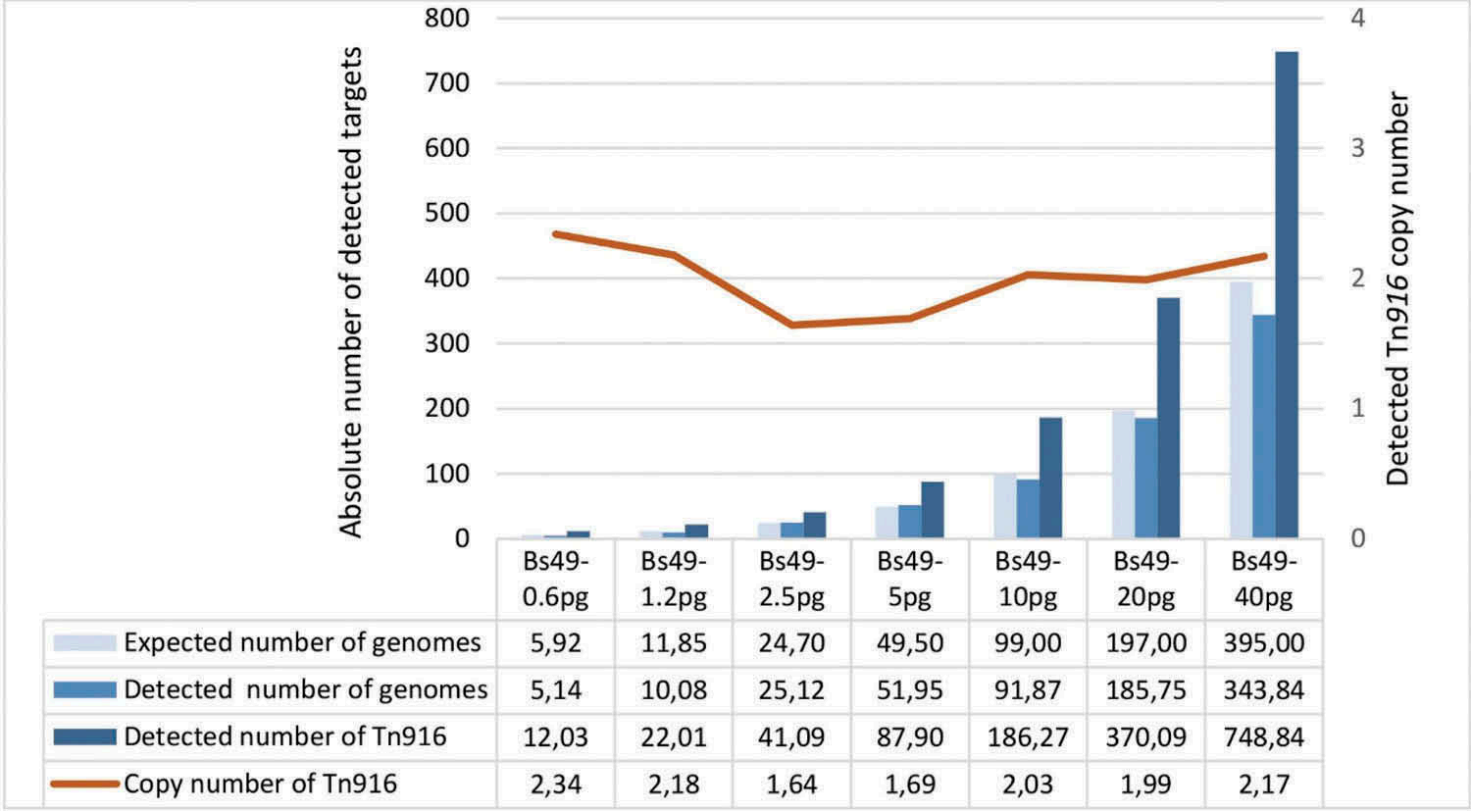
An illustration of the number of copies of *amyE* and *IntTn* and *XisTn* regions detected in *B. subtilis* BS49. *B. subtilis* BS49 has one copy of *amyE* and two copies of Tn*916* that are represented by *IntTn* and *XisTn* regions. The light blue bars show the theoretical single copy gene number of a 4.2 MB genome as calculated by QuantaSoft. Linearity was maintained across an increase of DNA concentration by two folds with the lowest input of 0.6 pg/µl and the highest input of 40 pg/µl.

Conventional PCR was conducted with primers listed in Table 2 and depicted in Figure 1S (see supplementary data). All reactions were performed in a final volume of 25 μl containing 12.5 μl of 2 x Dream Taq Green PCR master mix (Thermo Scientific, USA), 1 μl each of the forward and reverse primer (20 μM), 2.5 μl DNA sample, and 8 μl water. The PCR conditions were: initial denaturation at 94°C for 10 min, 30 cycles of 94°C for 1 min, 56°C for 1 min and 72°C for 2 min, and a final extension step at 72°C for 10 min. A volume of 5 μl was analyzed with a 1-KB DNA ladder on a 1% agarose gel containing GelRed™ (Biotium, USA) for visualization of the amplicon.

**Table 2.** Sequence of primers and probes used in the study.

Target	Forward primer	Reverse primer	Probe sequence	Amplicon size	Label (chlorophore)	Annealing temperature
*Tet(M)*	GGT TTC TCT TGG ATA CTT AAA TCA A	CCA ACC ATA CAA TCC TTG TTC AC	ATG CAG TTA TGG AAG GGA TAC GCT A	88bp	HEX/FAM	56℃
*amyE B. subtilis*	TGC AGA CGG AAT TTA CAC	CCG AGT CAT TAT ATA AAC CA	ACG GAT ACA ACC AAC GCA AA	146bp	HEX	56℃
Circular Intemidate (CI)ddpcr	CGT GAA GTA TCT TCC TAC A	GAC CTT GAT AAA GTG TGA TAA	AAT ACT CGA AAG CAC ATA GAA TAA GGC	167bp	FAM/HEX	56℃
*intTn* and *xisTn* regions	ATA CTC CCA TAC AGT CAA TAG TCC	AGT TCC ACC CCT GCA TGG	CCG TCG CAGGCA ATG AGT ATG GCT	88bp	FAM	56℃
*amyE* *S. sanguinus*	GGC GGA TGT CTA GGA GTT TAT C	TGG ATT GCC TTG CGT CTT	TTG GGC AAA TTC TCC GCT AAT GCC	67bp	FAM	56℃
*amyE* S. *oralis*	GGC ATC ATA GTC TGT ACC TGT G	AAC GGC TGG ACT CAC TTT AC	ACC AGT GCC AGT GGA AGT CAT TGT	96bp	FAM	56℃
*amyE* S. *mitis*	GCA TCC AAG CGG AAA CC	GAC CTA GAC TTT AAA CAT CCT GAA	TTT CCA TGA ACC AGT CAG CCC AGT	98bp	HEX	56℃
*amyE* S. *gordonii*	ATA AAT ACC AGA GCG TCG ACT T	CTA CTG CTA TTT CTG AAC CCT TTA TG	CAG TTC CAG TGA AAT GAT ACC AAT GCC A	149bp	FAM	56℃
*amyE* *E. faecium*	GAT TCG GAA CGA TGG AAG AT	GCG ATA CGG GCT TTC TTT AG	TTC AAA CCA TTG ATG CTG ATC CGA A	148bp	HEX	56℃
Circular Intermediate (CI) PCR	CGT GAA GTA TCT TCC TAC A	AC CTT GAT AAA GTG TGA TAA	N/A	166bp	N/A	56℃

**Table 3.** Accession numbers and genetic regions of the reference gene *amyE.*

Bacterial species	Accession number	*amyE* genetic region
*B. subtilis*	NZ_LN680001	327604..329583
*E. faecium*	CP012522.1	1785531..1787153
*Streptococcus sanguinis*	CP000387	1041272..1042738
*Streptococcus oralis*	FR720602	723983..725431
*Streptococcus mitis*	FN568063	702427..703881
*Streptococcus gordonii*	CP000725	1119068..1120519

### Genetic linkage between tet*(M)* and intTn and xisTn

In order to analyze the genomic proximity between *tet*(M) and *intTn* and *xisTn*, indicating that the genes are located on the same genetic element, linkage analysis by a duplex ddPCR was carried out. Intact bacterial cells were boiled for 5 min in molecular grade water to obtain a DNA template. The concentration of the DNA was determined using the Qubit 3.0 fluorometer (Life Technologies) and accordingly the optimal DNA amount was used for further analysis. The digestion reactions were performed in a 20 μl reaction mixture, which contained 2.5 μl of the bacterial DNA, 10 U of restriction enzyme, 2 μl of 10 x buffer, and water. The digestion was conducted at 37°C for 4 hrs prior to inactivation at 80°C for 20 min. The restriction enzymes used were *Bsu*RI (which does not cleave Tn*916* between *tet*(M) and *intTn* and *xisTn* genes) and *Hinc*II which cuts Tn*916* between the *tet*(M) and *xisTn* at position 14,934 bp in U09422.1. The *Hinc*II enzyme was used as a control for the genetic linkage analysis. The initial linkage between *tet*(M), *intTn*, and *xisTn* was automatically calculated by the Quantalife™ software as a ‘linkage’ score. This is the estimate of the total number of molecules (copies/µl) in the assay that contain fragments on which the two targets are physically linked. The linkage percentage is calculated by normalizing the linkage score for differences in DNA input between the two assays as described by Roberts et al. [31]. The linkage percentage (L%) between *tet*(M), *intTn* and *xisTn* was calculated as follows: L% = (2λ*_tet_*_(M) *intTn*/*xisTn* regions_/(λ *_tet_*_(M)_*+* λ *_intTn_*_/*xisTn* regions_)) ×100, where L% is the normalized linkage score, λ*_tet(M), intTn_*_/*xisTn* regions_ is the concentration of *tet*(M) and *intTn* and *xisTn* contributed to the *tet*(M)- *intTn* and *xisTn* genes droplets linkage, λ *_tet_*_(M)_ is the average number of *tet*(M) copies per one droplet and λ *_intTn_*_/*xisTn* regions_ is the average number of *intTn* and *xisTn* genes copies per one droplet.

### Evaluation of amyE, intTn, and xisTn genes as a representative gene and representative region for genome CN and *Tn*916 CN, respectively

The known genome size of *B. subtilis* BS49 and different amounts of DNA inputs based on Qubit 3.0 flourometer (Life Technologies) readings were used to evaluate the suitability of *amyE* as a representative gene for detection of the genome CN in ddPCR.

The formula used to calculate the expected genome CN of *B. subtilis* BS49 with a DNA input ranging from 0.6 pg to 40 pg in the ddPCR reaction mixture is as follows: Genome equivalents/copies = A/HGW where A is the input DNA concentration and HGW is the genome weight of the bacterial genome that was calculated according to the genome size in Mb multiplied by 0.001096.

In addition, the *intTn* and *xisTn* in *E. faecium* OrEc1 and *B. subtilis* BS49, which harbor five and two copies of Tn*916* respectively, were evaluated for being a representative region in Tn*916* for determination of the CN of Tn*916*-Tn*1545* like elements by ddPCR. The primers and probes used for the *amyE* and the *intTn* and *xisTn* genes were designed and labeled with either FAM or HEX as listed in Table 2.

### Calculation of the CN of *Tn916-*Tn1545-like elements and their CR by ddPCR

The QX200^TM^ Droplet Digital ^TM^ PCR system (Bio-Rad, Pleasanton, CA) was used in the current study to determine the CN of Tn*916*-Tn*1545-*like elements in the genome. In addition, the CR of Tn*916*-Tn*1545-*like elements that formed the CI in the *B. subtilis, E. faecium* and *Streptococcus* species populations were evaluated. The primers and probes used in the ddPCR assays in this study are listed in Table 2. The reaction mixture for CN experiments consisted of 10 µl ddPCR ^TM^ Supermix for Probes (No dUTP), 1 µl of 20 x *intTn*/*xisTn* regions (target gene) primers and probes, 1 µl 20 x of *amyE* primers and probe, 0.5 µl restriction enzyme (5 Units per reaction), 8 µl water, and 60 pg DNA template.

A total volume of 21 µl of the reaction mixture was transferred into the sample well of the cartridge, and 70 µl of droplet generation oil was applied to the correspondent oil well prior to placing the gasket over the cartridge and transferring it into the droplet generator. After droplet generation, 40 µl of the sample emulsion was transferred into a 96-well PCR plate (Eppendorf, Germany) and then sealed with pierceable foil (Bio-Rad). PCR amplification was done in a C1000^TM^ Thermal cycler (Bio-Rad). In all experiments, a non-template control (NTC) and positive controls were used to rule out any primer dimer or contamination issues. The amplification parameters consisted of an initial activation step at 95°C for 10 min followed by 40 cycles of 95°C for 30 s, and varying the annealing temperature (depending on the primers annealing temperatures as listed in Table 2) for 30 s. An additional inactivation step at 98°C for 10 min was used at the end of the cycles. The temperature ramp was set to 2°C per second and the lid was heated to 105°C. Upon completion of the PCR, the 96-well plate was transferred to the QX200 Droplet Reader (Bio-Rad) and the generated data were analyzed using the QuantaSoft software version 1.7.4.0971 (Bio-Rad). The threshold to distinguish positive droplets from the negative ones was set for each reaction automatically by the software if not stated otherwise. If needed, further analysis of the data was done using the QuantaSoft^TM^ PRO software (version 1.0).

The CN of Tn*916*-Tn*1545-*like elements per bacterial genome was calculated by using the ratio between the Tn*916* target region, that is *intTn* and *xisTn* and the single copy reference gene (*amyE*). For strain specificity, the variable region of the reference gene was used to design species-specific primers and probes for the studied species (Figure 2S in the supplementary data). The accession numbers of the *amyE* used in the current study are given in Table 3. In the CR experiments, the reaction mixture was prepared as described above with the exception of the primers and probes used, which in this case, only produced a signal if and when the element was in the circular form. The sequence of the primers, probes, product size and annealing temperatures are shown in Table 2.

The CR was measured by calculating the percentage of the CI molecules detected within the bacterial population, that is the number of detected copies of CI molecules in the bacterial population to the number of bacterial genomes represented by the *amyE* CN in the same population. Based on screening for the presence of CI by conventional PCR, seven oral *Streptococcus* strains; *S. oralis* (*n* = 4), *S. mitis* (*n* = 2), and S. *gordonii* (*n* = 1) were selected for the CR analysis. In addition to investigate the CR, this study also assessed the effect of varying the tetracycline concentration below the MIC levels on the excision of Tn*916*-Tn*1545-*like elements in the control strains, that is *E. faecium* and *B. subtilis*, and the oral *Streptococcus* strains.

It has been recently reported in *B. subtilis* that Tn*916* can replicate autonomously [20]. In order to determine whether the element in our strains was replicating autonomously we compared the ratio of detected CI to the detected copies of bacterial genome, represented by *amyE* CN.

### DNA sequencing of the promoter region upstream of tet*(M)*

DNA Sanger sequencing was used to investigate the DNA sequence of the promoter region upstream of *tet*(M) in oral streptococci. In brief, two primers (Table 2) were designed to yield a PCR fragment of 595 bp that covers the promoter region upstream of *tet*(M). The PCR fragment was subjected to BigDye terminator v 3.1 (Thermo Scientific) cycle sequencing prior to DNA sequencing by capillary electrophoresis using the SeqStudio sequencing platform (Thermo Scientific,). Sequencing data were aligned against the wild type Tn*916* using the Lasergene Molecular Biology Suite software (DNASTAR, USA).

## Results

### Bacterial strains

The 10 oral *Streptococcus* species. included in this study were identified by MALDI-TOF as follows: *S. mitis* (*n* = 2), *S. sanguinis* (*n* = 2),* S. oralis* (*n* = 5), and *S. gordonii* (*n* = 1).

### Evaluation of amyE as a representative gene for genome CN by ddPCR

The accuracy and sensitivity of using the *amyE* as a reference gene for detecting genome CN by ddPCR was achieved by analyzing varying concentrations of *B. subtilis* BS49 DNA (obtained from cultures that were cultivated in the absence of selection pressure). *B. subtilis* BS49 is known to harbor two copies of Tn*916* [26] and, as shown in Figure 1, the detected CN of Tn*916* using *amyE* as a reference gene over a two-fold increase in DNA concentration was on average 2.00, SD 0.24. The detected CN of Tn*916* measured up to the expected theoretical CN even at a low DNA input of 0.6 pg/µl.

### Determination of the CN of *Tn*916-*Tn*1545 -like elements

**Figure 2.**
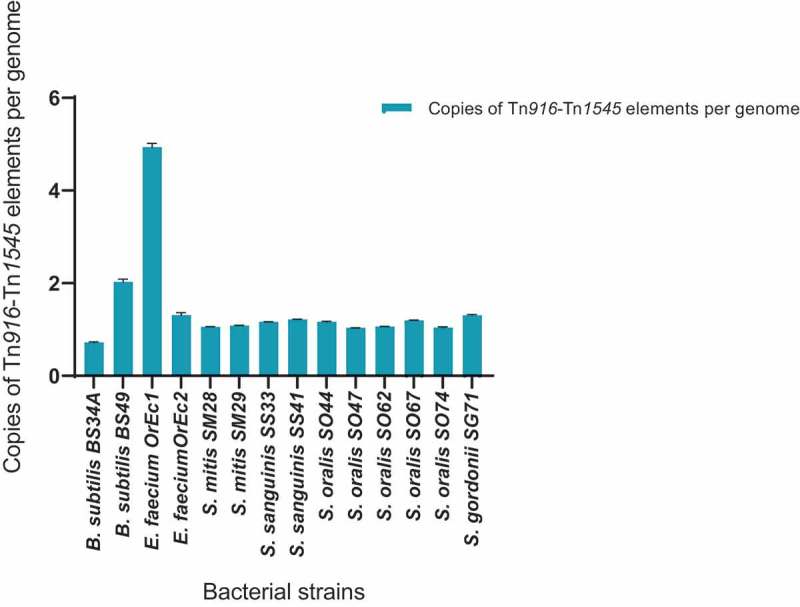
A graphical illustration of the copy number of Tn*916*-Tn1545 family detected in bacteria involved in this study. The bars represent the copies of the Tn*916*-Tn*1545* family per bacterial genome. The error bars represent 95% confidence intervals.

Prior to determining the CN of Tn*916*-Tn*1545-*like elements in *Streptococcus* species, we used previously published genome data of *B. subtilis* BS34A and *B. subtilis* BS49 (which contain one and two copies of Tn*916*, respectively) [26] and *E. faecium* OrEc1 and *E. faecium* OrEc2 (sequenced transconjugants produced in our laboratory which contains five copies and one copy of Tn*916*, respectively) to validate our ddPCR CN determination assay. In *B. subtilis* BS49 and *E. faecium* OrEc1 the observed number of Tn*916* corresponded to the expected CN (2 and 5 respectively). In *B. subtilis* BS34A, the observed CN was 0.72 as more *amyE* were detected than *intTn/xisTn*. The validated CN determination assay was used to screen a panel of 10 oral streptococci for determination of the CN of Tn*916*-Tn*1545*-like elements. Figure 2 shows that all the tested oral streptococci strains harbored only one copy of a Tn*916*-Tn*1545*-like element.

### Linkage between tet*(M)* and intTn and xisTn

**Figure 3.**
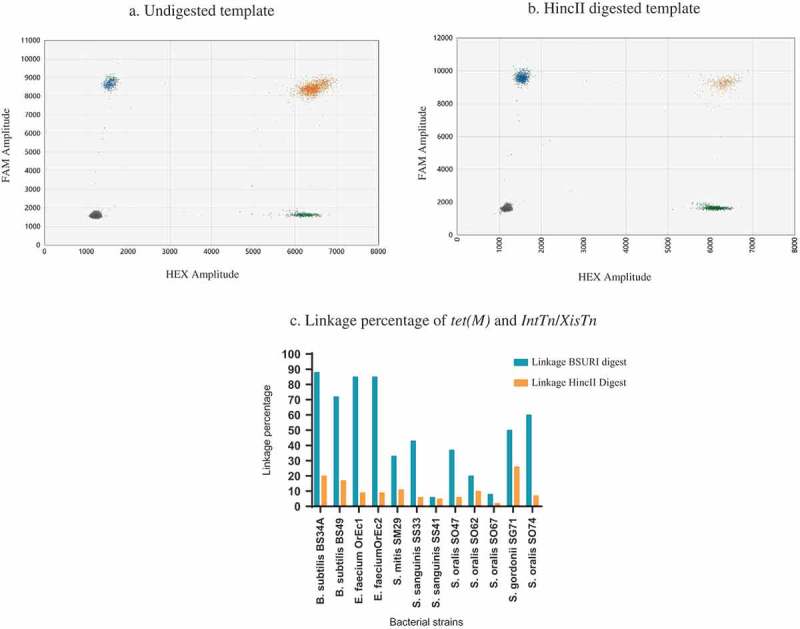
An illustration of Linkage between *tet*(M) and *IntTn*/*XisTn* regions. 3(a, b) show 2-D amplitude plot in which each axis represents the amplitude axis of either FAM or HEX. The blue droplets represent FAM targets (*tet(M))*, the green droplets represent HEX targets (IntTn/XisTn regions), the orange droplets are these that contain both FAM and HEX, and the gray represents the droplets with no target molecules. Image 3(a) shows the droplets distribution of undigested *B. subtilis* BS49 whereas as image 3(b) illustrates the target distribution when the template is digestion with *Hinc*II which cut between *tet(M*) and IntTn/XisTn regions in Tn*916*. 3C shows linkage percentage between *tet*(M) and IntTn/XisTn regions. The blue bars represent the linkage percentage between *tet*(M) and IntTn/XisTn regions. The orange bars show the drop of the linkage percentage when the two targets have been physical separated by restriction enzyme digestion.

The linkage percentage between *tet*(M) and *intTn* and *xisTn* regions, which represents the percentage of droplets containing both targets suggesting that they are physically linked on the same fragment of DNA. This linkage percentage ranged from 88% to 6% in the bacterial cells that were digested with *Bsu*RI as shown in Figure 3(c). In the control samples that were digested with *Hinc*II which not only cuts between the *tet(M*) and the *intTn* and *xisTn* genes but produces six fragments in Tn*916*, we observed a 3–10-fold drop in linkage (ranging from 2–26%). Figure 3(a, b) illustrates that the reduction in droplets that contain the double targets; *tet*(M) and the *intTn* and *xisTn* genes when digested with the two targets are physically delinked.

### The CR of *Tn*916-*Tn*1545-like elements

**Figure 4.**
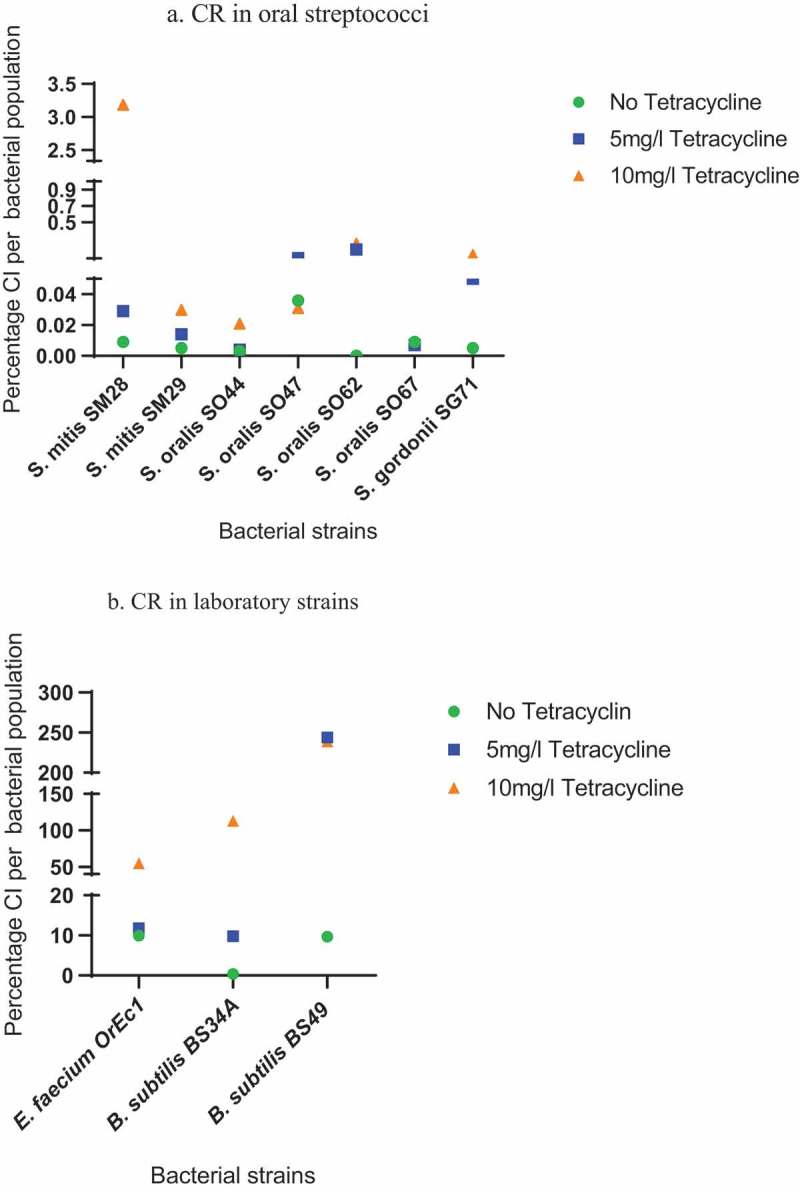
An image indicating percentage of CI per bacterial population. (a) CR in *E. faecium* OrEc1, *B. subtilis* BS34A, *B. subtilis* BS49. (b) CR in oral streptococci. The green circles represent the CR in the absence of tetracycline whereas the blue squares and the orange triangles represent the CR in the presence of 5 mg/ml and 10 mg/ml tetracycline, respectively.

The CN of the circular form of Tn*916*-Tn*1545*-like elements and the CN of *amyE* in the bacterial population were used to calculate the CR, that is the percentage of CI molecules detected within the bacterial population. Based on our findings, the values of CI vary among the bacterial species. In the selected clinical oral *Streptococcus* species, that is *S. oralis, S. sanguinis*, and *S. mitis*, the CR of Tn*916*-Tn*1545*-like elements was influenced by the presence and concentration of tetracycline. In the absence of tetracycline, the CR ranged from 0% to 0.036% while in the presence of 5 and 10 μg/ml tetracycline, the observed CR ranged from 0.004% to 0.17% and from 0.008% to 3.19%, respectively (Figure 4(a)). Interestingly, in *E. faecium* OrEc1, *B. subtilis* BS34A and *B. subtilis* BS49, the levels of CI were higher than in oral streptococci and influenced by the presence and concentration of tetracycline. In the absence of tetracycline, the observed CRs were, 9.9%, 0.4%, and 9.7% for *E. faecium* OrEc1, *B. subtilis* BS34A and *B. subtilis* BS49, respectively. In the presence of 5 μg/ml tetracycline the detected levels of CI increased to 11.8%, 9.8%, and 244% for *E. faecium* OrEc1, *B. subtilis* BS34A, and *B. subtilis* BS49, respectively (Figure 4(b)). When *E. faecium* OrEc1 was cultivated in the presence of 10 μg/ml tetracycline, it was observed that the percentage of CI molecules detected within the bacterial population exceed 50%. In both *B. subtilis* BS34A and *B. subtilis* BS49, the percentage of CI molecules detected within the bacterial population were 113% and 239%, respectively, exceeding the number of bacterial genomes that were detected in the assay.

### DNA sequencing of the promoter region upstream of tet*(M)*

The DNA sequencing results show distinct 58 bp deletions in two *S. oralis* strains, a 27 bp and 12 bp deletion in other two *S. mitis* strains, and multiple SNPs in the upstream of *tet(M)* in the other oral *Streptococcus* strains included in the CR experiment compared to the wild type sequence of Tn*916*. The deletions effectively removed the predicted large terminator structure responsible for transcriptional attenuation (Supplementary data, Figure 3S).

## Discussion

Antibiotic resistance in oral streptococci is an ever-growing problem [32]. Advances in molecular biological techniques and detection methods of resistance genes, have increased our knowledge of factors contributing to the propagation of MGEs carrying resistance genes in bacterial populations. The CN determination of MGEs carrying resistance genes in oral streptococci is of particular interest, as high CN might influence the propagation and spread of resistance due to availability of more than one element in any given genome. Furthermore, the presence of more than one copy of MGEs might influence bacterial biological fitness [33] and hence the reversibility of resistance. Our attempt to determine CN of MGEs, such as Tn*916*-Tn*1545*-like elements, is part of ongoing work to determine the biological cost of these elements in oral streptococci. This work has led to the development of an assay that can easily and accurately determine the CN of Tn*916*-Tn*1545*-like elements in *E. faecium, B. subtilis* and oral streptococci using ddPCR. We tested the sensitivity and reproducibility of our assay by analyzing varying amounts of input DNA that contained a predetermined number of target regions per genome. Our results illustrate that ddPCR is a sensitive and highly specific tool that can be used to determine CN of MGEs. The reproducibility and precision, even at very low input DNA concentration (0.6 pg/µl) is promising as it allows for analysis of samples with low DNA target concentrations and may be applicable therefore to analysis of bacteria directly from saliva samples and other body fluids.

Four sequenced bacterial strains; *B. subtilis* BS34A (NZ_LN680001.1), *B. subtilis* BS49 (NZ_LN649259.1) *E. faecium* OrEc1, and *E. faecium* OrEc2 (unpublished data) were used to determine the accuracy of ddPCR in detecting multiple copies of Tn*916*-Tn*1545*-like elements. In *B. subtilis* BS49, *E. faecium* OrEc1, and *E. faecium* OrEc2, we were able to accurately detect the expected number of elements using *amyE* as a chromosomally located, single copy, reference gene. In *B. subtilis* BS34A however, the ratio between Tn*916*-Tn*1545*-like elements (represented by the *intTn*/*xisTn* genes) and the reference gene *amyE* was below one copy (approximately 0.75). The lower ratio may be explained by the chromosomal positioning of the two targets in relation to the origin of replication. In *B. subtilis* BS34A, the *amyE* gene (327,604–329,583 bp) is situated closer to the origin of replication in comparison to Tn*916* which is in position 1,886,552–1,904,583 bp. The closer proximity of *amyE* to the origin of replication may result in more targets of the reference gene due to the occurrence of multiple replication forks within a cell prior to cell division, as has been previously reported in *B. subtilis* [34].

A few studies have reported the presence of more than one copy of Tn*916*-Tn*1545*-like elements in clinical strains [35–37]. Rice et al. (2005) reported that the presence of multiple copies Tn*916*-Tn*1545* elements in clinical strains is rare [38]. The current study supports such findings as all our clinical oral streptococci contained one copy of Tn*916*-Tn*1545*-like elements.

The ability to detect linkage between two genetic targets; the likelihood that two genetic targets are in physical proximity of each other, is fundamental to our understanding of the likelihood of horizontal gene transfer of resistance genes, especially if the linked genetic target to the resistance determinants is responsible for mobility. Several Tn*916*-Tn*1545-*like elements that confer resistance to more than one antibiotic have been reported [2,36,39–40]. The ability to determine whether or not these resistance genes are on the same mobile element will shed light on the prevalence of these resistance determinants and the likelihood for co-transfer. In our study, we illustrate that ddPCR can be used to determine the linkage between *tet*(M) and *intTn* and *xisTn* genes of Tn*916* in *B. subtilis* BS49, *E. faecium*, and in oral streptococci. Whilst most Tn*916*-Tn*1545* elements share an extremely high degree of sequence homology we acknowledge that subtle variations in the DNA sequence of the genetic target for ddPCR will affect the efficiency of the reaction. This can be overcome by using multiple sets of primers to detect the element itself and previously published primers for various resistance genes based on conserved regions within these genes.

The ratio between CI and the number of bacterial genomes present in the bacterial population was used to investigate the CR of the elements. The basal rate of CI formation varied between and within species. In *B. subtilis* BS34A, *B. subtilis* BS49, and the *E. faecium* OrEc1, the CR ranged from 0.4% to 9.9% in the absence of tetracycline. In oral *Streptococcus* strains, the CR in the presence of tetracycline (5 μg/ml and 10 μg/ml) was observed to range from 0.004% to 3.19% (Figure 4(a)). In contrast, the CR in *B. subtilis* and *E. faecium* strains in the presence of the same tetracycline concentrations showed higher numbers of CI in the bacterial population. It can be hypothesized that the observed lower CR in oral streptococci may be due to the fact that these are clinical isolates containing Tn*916*-Tn*1545*-like elements that have evolved a mechanism to reduce the level of excision to reduce any fitness cost associated with acquisition of these elements, a theory that requires further investigation for these strains. Sequencing of DNA upstream of *tet*(M) shows several SNPs and deletion when compared to the wild type sequence of Tn916. The presence of deletions results in the removal of the predicted large terminator structure responsible for transcriptional attenuation upstream of tet(M). The removal of the large terminator structure would suggest that these isolates would not respond to tetracycline in the same way as it is hypothesized for the wild type Tn*916*.

Another possible explanation for the low CR of Tn*916*-Tn*1545* in oral streptococci is that *tet*(M) is not present on the Tn*916*-Tn*1545* like elements in some strains (e.g. *S. sanguinis* SS41 in Figure 3(c)).

In both *B. subtilis* strains, cultivation in the presence of 10 μg/ml tetracycline resulted in levels of CI that were higher than the number of bacterial genomes detected. This suggests that at this concentration of tetracycline, Tn*916* is undergoing autonomous replication; a phenomenon that has been recently demonstrated [20]. The observed increase in CI presumably occurred because tetracycline could result in increased mobility of Tn*916* as shown by Scornec et al. [41]. Introduction of even higher concentrations of tetracycline did not result in more CR in our bacterial populations, but rather we observed a decrease of CI number in the bacterial population where the concentration of tetracycline reached higher values, although still under the MIC values for these resistant strains (Table 1). The latter observation could be attributed to an overall effect on protein synthesis thus leading to slower or nearly diminished replication [12].

It is reasonable to assume that the more copies of the Tn*916*-Tn*1545* family present in the genome, the more CI molecules would be present in the bacterial cell. This assumption was true when CI levels in *B. subtilis* BS49 strain; containing two copies of Tn*916*, were compared to CI levels in *B. subtilis* BS34A, which harbors only one copy of Tn*916*. This is consistent with findings from previous studies [42].

In this study, we demonstrate that ddPCR can be used to study CN and CR of the Tn*916*-Tn*1545* family in oral streptococci with and without the presence of antimicrobial challenge. In addition to detection of the CI, we have also demonstrated that ddPCR can be used to detect an increase in the CN of the target molecule compared to another, as would happen if the CI of Tn*916* was autonomously replicating. The minimal skills requirements, and flexibility, requirements for small amounts of DNA sample, and good reproducibility illustrate the potential that ddPCR carries for the advancement of studying MGEs like Tn*916*-Tn*1545* family and antibiotic resistance.
