# Supplementary material for: Determination of copy number and circularization ratio of Tn916-Tn1545 family of conjugative transposons in oral streptococci by droplet digital PCR
Source: J Oral Microbiol. 2018 Dec 6;11(1):1552060. doi: 10.1080/20002297.2018.1552060 (PMC6292373; doi:10.1080/20002297.2018.1552060)
Supplement: Supplemental Material [file ZJOM_A_1552060_SM2528.docx]

Supplimentary Data

Figure 1S. A non scaled graphical representation of primer binding sites in Tn916 where CI-Rv (position 51bp) and CI-Fw (position 17928bp) representing a 167bp amplicon of the circular intermediate (CI); tetMFw (position 13511bp) and TetM Rv (position 13559bp) represent the 88bp *tet*(M) gene amplicion; IntFw (position 16185bp ) and Xis-Rv (position 16271bp) representing a 88bp amplicon.

S.Oralis_α-amylase ------------TTATTTTGCCGCCCAGACACTGACTGAACCCGCTGCTACTGGAAATTC 48

S.sanguinis_α-amylase ---ATGAAGAGGAAAATTTATATGGAAAACCAAACC-T-----------TAATGCAGTAT 45

S.mitis_α-amylase ATGCAAAATCAAACACTTATGCAATACTTTGAATGG-----------------TATCTGC 43

S.gordonii_α-amylase --------ATGAAAAACCAAACTTTAATGCAGTATTTT-----------GAATGGTATCT 41

S.Oralis_α-amylase TCCATAACCTTCAGCATTGATTGTAACTTGTGCTGGATGATTTTCAAGGAGGTCAATAAA 108

S.sanguinis_α-amylase TTTGAATGGTATCTGCCAGATGACGGTCAGCATTGGAATCGCTTAGCGGAAGATGCACCA 105

S.mitis_α-amylase CCCACGACGG--CCAGC--------------ACTGGACGCGTCTAGCTGAAGATGCTCAA 87

S.gordonii_α-amylase TCCAGATGATGGCCAGC--------------ATTGGAATCGTTTAGCAATGGATGCGCCA 87

S.Oralis_α-amylase GGTTTGTTCAGCCCATTCTTGCCCGACAAACATAGCCTTGCTGTTTTCTTGGTCATTTGA 168

S.sanguinis_α-amylase AACTTAGCAGCGAAAGGAATTCGCAAAGTCTGGATGCCGCCGGCTTTCAAGGGAACGGGC 165

S.mitis_α-amylase CACCTAGCTGATCTCGGCATTAGTCATGTCTGGATGCCCCCAGCTTTTAAGGCAACCAAT 147

S.gordonii_α-amylase AATCTAGCAGCCAAAGGAATTAAAAAAATATGGATGCCTCCAGCTTTTAAAGCAACTGGC 147

S.Oralis_α-amylase GATAAGGACA---GCG-----ATTGGGGATTGATGTTCA------------GCACCTGAA 208

S.sanguinis_α-amylase TCTAATGACGTCGGCTATGGTGTTTATGACCTCTTTGATTTGGGAGAGTTCGACCAAAAA 225

S.mitis_α-amylase GAAAAAGATGTAGGCTATGGTGTCTATGACTTATTTGACTTAGGAGAATTTAATCAAAAA 207

S.gordonii_α-amylase TCAAATGATGTTGGCTATGGCATTTACGATTTATTTGATTTAGGAGAGTTTGATCAAAAA 207

S.Oralis_α-amylase CGTACCCATCCGATACAGTTGGCATCGTCAAAGTAGTCTGTTTGCTCTCCATAGGCCATG 268

S.sanguinis_α-amylase GGGACCGTCCGCACAAAGTATGGATTGAAGG--AAGAATACCTCCGAGCGATTGAAGCGC 283

S.mitis_α-amylase GGAACTGTCCGCACCAAGTATGGTTTTAAAG--AAGACTATCTTCAAGCCATTCAAGCCC 265

S.gordonii_α-amylase GGGACAGTTCGGACTAAGTACGGATTCAAAG--ACGAATACCTTCAAGCAATCCAAGCAC 265

S.Oralis_α-amylase TCTTTTCGGATGGTTAGGAGACGATCAAGAACTTCTCTGAAATCTTGTTGAGCAAATTGC 328

S.sanguinis_α-amylase TTAG---------CCAAAACGGTATCGAAGCTATTGCAGATGTGGTTCTCAATCACAAGG 334

S.mitis_α-amylase TAAA---------AGCGCAGGGAATCCAGCCCATGGCCGATGTGGTACTCAACCACAAGG 316

S.gordonii_α-amylase TTAA---------AGATAATGGAATCGATCCAATTGCTGATGTCGTTTTAAATCATAAAG 316

S.Oralis_α-amylase CCTGAAATGCCGTAATAGTCTCCATAAAAGACACATGGAAGACCTTGCTCACGAAGAAGG 388

S.sanguinis_α-amylase CCGCAGCTGACTATAAAGAGCGCTT-----TACCGTTGTTGAAGTTGATCCTAACAACCG 389

S.mitis_α-amylase CTGCAGCCGATCACATGGAAGCCTT-----TCAGGTTATCGAAGTGGATCCTGAAGATCG 371

S.gordonii_α-amylase CTGCAGCCGATGGAACTGAAACATT-----TACGGTTATTGAAGTAGATCCAAATGACCG 371

S.Oralis_α-amylase ATAAGGGCATAGGCTGCTGGCTTAAACCATTCTTCAAC----AGTAGACTCAAGGGCCTG 444

S.sanguinis_α-amylase -------CACAAAGGTCTTGTCAGAACCTTTCGAGATTAAAGGCTGGACTAAGTTTGTCT 442

S.mitis_α-amylase -------TACAGTTGAACTTGGAGAACCCTTCACCATCAATGGCTGGACTAGTTTTACCT 424

S.gordonii_α-amylase -------TACTACTGCTATTTCTGAACCCTTTATGATTAAAGGATGGACTCACTTTACTT 424

S.Oralis_α-amylase TCC---------TCGTTGAGTATCATGGTTGTCAACGAAAGTGACAGCCTTGTCAGGCTT 495

S.sanguinis_α-amylase TCCCAGGCCGCAAAAAAGCCTACAATGACTTTGAATGGCACTGGTACCACTTC------- 495

S.mitis_α-amylase TCGATGGCCGCCAAGATACCTACAATGACTTCCACTGGCATTGGTACCACTTC------- 477

S.gordonii_α-amylase TTCCAGGAAGAAACAAGCAATATAATGATTTTGAATGGCATTGGTATCATTTC------- 477

S.Oralis_α-amylase GAGTTCAACCAAGCTATCAGTAAAGATAG---TACGAAGGTCGTAGCTTGCTCCAGCCTG 552

S.sanguinis_α-amylase --ACCGGCACTGACTATGATGCCAAAAACAACAAGTCAGGCATTTTTCTCATCCAAGGGG 553

S.mitis_α-amylase --ACAGGTACAGACTATGATGCCAAACGTCGTAAGTCTGGGATTTATCTGATCCAAGGGG 535

S.gordonii_α-amylase --ACTGGAACTGACTTTGATGCAAAAAGTCGACGCTCTGGTATTTATTTAATTCAAGGTG 535

S.Oralis_α-amylase ACTAGCTTCAAAGAGGTTTTGGTGGAGTCGAACATCGACAAGGTCAAAACGTTCTTCTGT 612

S.sanguinis_α-amylase ACAATAAAGGTTGGGCAGATGATGAG----CTAGTGGACAACGAGAATGGTAACTACGAC 609

S.mitis_α-amylase ACAACAAAGGCTGGGCCAACGAGGAA----TTAGTCGATAACGAGAACGGAAACTACGAC 591

S.gordonii_α-amylase ACAATAAAGGTTGGGCAAATGATGAG----CTGGTAGACAGCGAAAACGGAAACTATGAC 591

S.Oralis_α-amylase TTTCTCAAGATAGTCTAGATTGGCTTCCTTGTCTGGATTCCAAAATTCCCCAAAAACATA 672

S.sanguinis_α-amylase TATCTCATGT------ATGCGGATATTGATTTCAAGCACCCCGAAGTCATCCAAAATCTC 663

S.mitis_α-amylase TACCTCATGT------ATGCTGACCTAGACTTTAAACATCCTGAAGTCATCCAAAATATC 645

S.gordonii_α-amylase TATCTAATGT------ATGCCGATCTAGATTTCAAACATCCAGATGTTATTAAAAATTTA 645

S.Oralis_α-amylase GAAATCTTGACCGTATTTTTCCTTCATATCACGGATGAAATTGCCCATAAAGAAGGAGTC 732

S.sanguinis_α-amylase TACGACTGGGCTCATTGGTTTATTGAAAGCACTGGTGTACATGGCTTTCG------ATTA 717

S.mitis_α-amylase TATGACTGGGCTGACTGGTTCATGGAAACGACTGGTGTAGCTGGTTTCCG------CTTG 699

S.gordonii_α-amylase TACGATTGGGCTAAATGGTTTATCGAAACTACTGGTATACAGGGCTTCCG------ATTG 699

S.Oralis_α-amylase GATGTGCTTAACGGCATCCAAACGGAAA------CCAGCCACACCAGTCGTTTCCATGAA 786

S.sanguinis_α-amylase GATGCTGTCAAGCACATCGATTCTTTCTTTATGAAAAATTTCATCCGCGATATTACTGAA 777

S.mitis_α-amylase GATGCAGTCAAACACATCGATTCTTTCTTTATGCGCAATTTCATCCGCGATATGAAGGAA 759

S.gordonii_α-amylase GACGCGGTTAAACATATAGATTCCTTCTTTATGGCAAACTTTATTCGAGATATTTTGCAA 759

S.Oralis_α-amylase CCAGTCAGCCCAGTCATAGATGTTTTGGATGACTTCAGGATGCTTAAAGTCTAGGTCAGC 846

S.sanguinis_α-amylase AAATACGGTGAAGATTTCTATGTTTTTGGGGAATTTTGGAATAGCGATGAGAAGGCCAAT 837

S.mitis_α-amylase AAATACGGTGACGATTTCTACGTTTTTGGTGAATTTTGGAACCCAGACAAGGAAGCCAAT 819

S.gordonii_α-amylase GAATATGGTGATGATTTCTATGTTTTCGGAGAGTTTTGGAATAATGATGAAGCTGCTAAT 819

S.Oralis_α-amylase ATACATGAGGTAGTC----GTAGTTACCGTTTTCGTTATCGACCAATTCCTCATTTGCCC 902

S.sanguinis_α-amylase AATGATTATCTAGAAAATATTGACTACCGCTTTGACCTAGTCGATGTTAAACTTCATCAT 897

S.mitis_α-amylase CTAGATTATCTTGAAAAAACAGAAGAACGCTTTGACCTTGTCGATGTTCGTCTCCACCAG 879

S.gordonii_α-amylase AATGACTACTTAGAAAATATTGACTATCGCTTCGATCTTGTCGATGTTAAATTGCATACT 879

S.Oralis_α-amylase AGCCCTTGTTGTCCCCCTGGATCAGGTAAATGCCAGACTTACGGCGCTTGGCATCATAGT 962

S.sanguinis_α-amylase AATTTATTTGATGCCAGCAAATCTGGAGCCGAC----TATGACCTACGAACTATTTTTGA 953

S.mitis_α-amylase AATCTCTTTGATGCTAGTCGAGCAGGTTCCAAC----TATGACCTTCGTGGCATTTTCAA 935

S.gordonii_α-amylase AATCTTTTTGAAGCAAGCCAAAAAGGTGAAGAA----TATGACTTGCGTACTATTTTTGA 935

S.Oralis_α-amylase CTGTACCTGTGAAG---TGGTACCAGTGCCAGTGGAAGTCATTGTAGATATCTTGGCGGC 1019

S.sanguinis_α-amylase CCAAACACTTGCAAAGAATCATCCTGAATCAGCTGTGACCTTTGTAGATAA--------- 1004

S.mitis_α-amylase AGATAGCCTGGTTGAACTCAAACCTGACAAGGCTGTGACATTTGTCGACAA--------- 986

S.gordonii_α-amylase TCATACTTTAGTAAAAAATCACCCAGAATCAGCTGTGACATTTGTAGAAAA--------- 986

S.Oralis_α-amylase CATCGAAAGTAAAGTGAGTCCAGCCGTTGATAGTAAAGGGCTCGCTTAGTTGAACAGTAC 1079

S.sanguinis_α-amylase TCACGATACTCAGAGAGGTCAGGCTTTGGAGTCTACCGTCGAAGAATGGTTCAAGCCGGC 1064

S.mitis_α-amylase CCACGATACACAACGAGGTCAGGCTCTTGAGTCTACCGTTGAAGAATGGTTCAAACCAGC 1046

S.gordonii_α-amylase TCATGATACTCAGCGTGGTCAAGCTCTTGAATCCACTGTAGAAGAATGGTTTAAACCGGC 1046

S.Oralis_α-amylase G----ATCCTCAGGATCCACTTCAATAACCTGAAA-GGCTTCCATATGATCGGCAGCAGC 1134

S.sanguinis_α-amylase GGCCTATGCTCTTATACTTCTAAGAGAAGCTGGATTGCCTTGCGTCTTTTATGGAGACTA 1124

S.mitis_α-amylase AGCCTACGCCCTCATTCTGTTACGCCAAAATGGCCTTCCATGTGTCTTTTACGGCGACTA 1106

S.gordonii_α-amylase AGCTTATGCTCTTATTCTACTTCGAGAGTCTGGCCTCCCATGTATTTTCTACGGAGACTA 1106

S.Oralis_α-amylase CT-TGTGATT----------------------------GAGCACCACATCAGCCATAGGT 1165

S.sanguinis_α-amylase CTATGGCATTAGCGGAGAATTTGCCCAAGAGAGCTTTCAAGAGCTACTGGATAAACTCCT 1184

S.mitis_α-amylase CTATGGGATTTCAGGGCAATATGCTCAACAAGATTTCAAAGAAGTCCTTGACCGCCTCCT 1166

S.gordonii_α-amylase CTATGGCATAAATGGTGAATTCGCTCAGCAAGATTTCCAAGAAGAGATCGACAAACTATT 1166

S.Oralis_α-amylase TGAATTCCCTGTGCCTTTAGGGCTTGAATGGCCTGAAGATAGTCTTCTTTAAACCCATAC 1225

S.sanguinis_α-amylase AGACATCCGCCTCAATCTAG----CCTATGGTGAGCAGACCGACTACTTTGACGATGCCA 1240

S.mitis_α-amylase AGCCATCCGAAAAGATTTGG----CCTATGGAGAGCAAACAGACTACTTTGATGACGCAA 1222

S.gordonii_α-amylase GGACCTTCGTCTAAACCTAG----CTTATGGTGAAGAGACCGACTACTTTGATGACCCTA 1222

S.Oralis_α-amylase TTGGTACGGACAGTCCCTTTTTGGTGAAATTCGCCTAGGTCAAAAAGATCGTAAACACCA 1285

S.sanguinis_α-amylase --------------ACTGTATTGGCTGGACTCGCCAAGGTATGGACGATGGTCAGCCAAT 1286

S.mitis_α-amylase --------------ACTGTATCGGTTGGGTACGTTCAGGTGCTGAAAATCAATCCCCAAT 1268

S.gordonii_α-amylase --------------ATTGTATCGGATGGACAAGGGCTGGTCAAGATGGTAGTCAGCCTAT 1268

S.Oralis_α-amylase TAGCCTACATCTTTTTCGTTGGTT---GCCTTGAAGGCAGGTGGC-----ATCCAGACAT 1337

S.sanguinis_α-amylase CGCTGTCCTTATCAGTAATGACCAAGCAACCAACAAATCCATGCTTGTCGGTCCAGAATG 1346

S.mitis_α-amylase CGCAGTCCTTATCTCAAATGACCAAGAAAACAGCAAGTCAATGTTTATCGGCCAAGAATG 1328

S.gordonii_α-amylase TGCTGTTTTAATTTCAAATGCTTCTGCTACTTCAAAACGAATGTATTTTGGCCAAGATTG 1328

S.Oralis_α-amylase GGCTGATACCAA-------GGTTTGCTAGGTGCTCTGCGT-CATTTGTTAGTCTAGCCCA 1389

S.sanguinis_α-amylase GGCTGGCAGAAAATTCAGTGACTATCTAGGCAACAGCTCTCAAATCGTAACTATTGACGA 1406

S.mitis_α-amylase GGCTAACCAAACTTTTGTAGATTTACTTGGAAATCACCAAGGTCAAGTTACAATCGATGA 1388

S.gordonii_α-amylase GTCAGGGCACGAGTGTTCCGACTACCTTGGTAATTGCCAAACAATTGTCACTATCGATGA 1388

S.Oralis_α-amylase GTGCTGGCCGTCATGAGGCAGATACCATTCAA--AGTATTGCATAAGTGTCTGATTTTGC 1447

S.sanguinis_α-amylase CCAA----G-GCTGGGGAGAATTTCCTGTGGAGGAAAAATCAGTTAGTGTCTGGAGTCTT 1461

S.mitis_α-amylase GGAA----G-GTTATGGACAATTCCCAGTCTCAGCAAGATCTGTAAGTGTCTGGGCAGCC 1443

S.gordonii_α-amylase AGAA----G-GCTGGGGAGTGTTCCCTGTTGAAGAACAATCTGTTAGCGTTTGGTCTATT 1443

S.Oralis_α-amylase AT---------- 1449

S.sanguinis_α-amylase AGATAA------ 1467

S.mitis_α-amylase AATACTATTTAA 1455

S.gordonii_α-amylase AAAAAATAA--- 1452

Figure 2S. The DNA sequences of *amyE* genes used for strain specificity. The variable region of the gene was used to design species-specific primers and probes for the studied oral streptococci species.

# Figure 3S: Alignment of DNA sequence of Tn*916*-Tn*1545* elements found in *S. oralis* (n=4), *S. mitis* (n=2) and *S. gordonii* (n=1) upstream of *tet(M)* in wild type Tn*916* (accession no. U09422.1) from *B. subtilis* BS34A. The depicted DNA sequence is the reverse complement DNA sequence starting at 11976 to 12093 bp.
